# Enabling Recovery Following Peer‐Perpetrated Sexual Violence: Lived Experiences of Young Women in Sweden

**DOI:** 10.1111/scs.70304

**Published:** 2026-08-08

**Authors:** Jenni Isaksson, Lina Palmér, Maria Lundvall

**Affiliations:** ^1^ Faculty of Caring Science, Work Life and Social Welfare, Department of Caring Sciences University of Borås Borås Sweden

**Keywords:** adolescent, caring science, gender‐based violence, phenomenology, qualitative research, quality of life, rape

## Abstract

**Introduction:**

Peer‐perpetrated sexual violence is very common in young adult relationships. In Sweden, one in four young women has experienced sexual violence, and it is a leading cause of mental health issues among young women today. Knowledge about how recovery is enabled for young women with experience of peer‐perpetrated sexual violence remains limited. The primary emphasis of caring is to support health and well‐being and to contribute to recovery; therefore, understanding the meaning of how recovery is enabled is essential when caring for these young women.

**Aim:**

The aim of this study is to describe the meaning of *enabling recovery* as experienced by young women following peer‐perpetrated sexual violence.

**Methods:**

A reflective lifeworld research approach was applied in this phenomenological study. Twelve young women, aged 17 to 25 years, participated in lifeworld interviews exploring the meaning of enabling recovery following peer‐perpetrated sexual violence.

**Results:**

The essential meaning of enabling recovery is described as: “By unveiling the hidden, the movement of life reclaims existence.” This is further described through the four constituents: Striking insight, A haven for rest, Existential safeness and Sharing one's story in a safe place. The enabling of recovery from peer‐perpetrated sexual violence emerges as a dynamic movement that involves both physical and existential aspects. It emphasises that reclaiming one's existence requires a movement in life that strives to enhance well‐being. Every element that contributes to this movement is interconnected, and no part can function in isolation from the others.

**Conclusions:**

This study provides an in‐depth understanding of how recovery is enabled following peer‐perpetrated sexual violence. It highlights the long‐term impact on young women's health and well‐being and emphasises the importance of a lifeworld‐led, recovery‐oriented caring approach that addresses both physical and existential dimensions. Enabling recovery is understood as an interconnected and dynamic process of meaning‐making, where uncovering lived experiences and supporting existential safeness and stillness help young women to reclaim their existence.

## 1 Introduction

The experience of sexual violence is widely recognised as one of the leading causes of numerous health issues for women [1, 2, 3, 4, 5, 6]. Every experience of abuse and violence is unique, but what makes the experience of sexual violence stand out is the embodiment of the abuse [7]. Earlier research has shown that sexual violence profoundly detaches women from themselves, from others and from the world [8]. This makes sexual violence uniquely harmful because it undermines trust in others and disrupts the expectations that are necessary for interpersonal functioning [9], creating severe barriers to well‐being and negatively affecting women's health.

Many young women experience sexual violence within their first sexual relationships and, in Sweden, sexual violence in peer relationships is relatively common [10]. In a national survey, 24% of women (aged 16–29) reported experiencing sexual coercion, and 19% reported experiencing physical violence by a peer during sexual activity [11]. Peer‐perpetrated sexual violence is defined in terms of the relationship in which the individuals involved are of similar age and social status, and the violence often occurs within a youth dating context.

From an existential and caring science perspective, recovery can be understood as part of a process that strengthens health and supports well‐being [12]. Health can be described as the experience of well‐being, encompassing a holistic perspective on the human being that acknowledges both physical and existential dimensions [13]. Health can be further understood as the connection between well‐being and the ability to engage in activities that give life meaning [12]. When health and well‐being are present, a sense of movement and capability arises. In contrast, illness is often associated with feelings of immobility, being trapped and having limited possibilities [12]. When recovery is understood as a process of regaining the ability to engage in life in a meaningful way, it becomes significant to understand how such recovery is enabled. This includes a view of humans as beings who, in some sense, can transcend their circumstances. Since humans are not seen as determined but always in process [12], this opens up the possibility of responding to our life circumstances, since existential freedom confers choice and agency on human life.

Being subjected to peer‐perpetrated sexual violence can be understood as a cause of illness in young women's lives. Following the abuse, young women find themselves in a state in which meaningful activities are no longer attainable [14]. This can be described as a disruption of their life movement; the experience of peer‐perpetrated sexual violence throws young women into an existential void where isolation, loneliness and silence take hold and everyday functioning becomes difficult to sustain [14]. Little attention has been paid to how recovery is enabled, and research on this phenomenon remains scarce.

It is essential to understand how recovery is enabled for young women who have been subjected to peer‐perpetrated sexual violence in order to develop and provide a caring environment that is genuinely supportive. The act of caring involves supporting and strengthening health processes and well‐being, even when a person's existence is threatened by illness. Therefore, this study aims to describe the meaning of *enabling recovery* as experienced by young women following peer‐perpetrated sexual violence in Sweden.

## 2 Methods

This study employs a Reflective Lifeworld Research (RLR) approach rooted in phenomenology and hermeneutics [15]. A qualitative, phenomenon‐oriented methodology was utilised in order to gain a deep understanding of the lived experiences of young women with a history of peer‐perpetrated sexual violence. As a methodological approach, RLR provides the researcher with a phenomenon‐oriented focus and an epistemological foundation throughout the research process, grounded in the principles of openness, flexibility and bridling [15]. Husserl [16] describes a phenomenon as something experienced by an individual, and the phenomenon explored in this study is enabling recovery following peer‐perpetrated sexual violence as experienced by young women.

### 2.1 Participants and Data Collection

Following Dahlberg, Dahlberg and Nyström [15], lifeworld interviews were used for data collection. Altogether, 12 interviews were conducted with young women who had experienced sexual violence from a male peer, friend or boyfriend. The inclusion criteria required participants to identify as young women, be between 16 and 25 years old, and have experienced sexual violence involving a perpetrator within the same age range. All participants were native Swedes from various regions across Sweden. At the time of the interviews, the young women were aged between 17 and 25. The interval between the abuse and the interview ranged from 10 months to 10 years. The experience of sexual violence ranged from a single isolated incident to recurrent abuse over several years within a relationship, with all perpetrators identified as young men. The average age at first victimisation was 14.

Lifeworld interviews focus on lived experiences and the meanings of the phenomena being studied. The interview process involved actively exploring the interviewees' understanding of the phenomenon. Open questions were posed to gather the lived experience of enabling recovery after experiencing peer‐perpetrated sexual violence and to encourage participants to reflect upon the phenomenon. The initial questions, with minor variations, were formulated as: “Can you tell me what helped you feel better after the assault?” and “What does recovery mean to you?” To maintain a reflective attitude throughout the interview, and to help participants deepen their understanding of the phenomenon, follow‐up questions like: “Can you tell me more?”, “How did that feel to you?” and “What did you do?” helped participants to describe their experiences. Being open to a phenomenon showing itself in new ways and, consequently, questioning one's understanding of that phenomenon provides flexibility throughout the interview and research process.

All 12 interviews took place in 2023. One was conducted by telephone, and the other 11 via encrypted Zoom. The average interview duration was 83 min, and all were transcribed verbatim by the first author.

### 2.2 Data Analysis

We explored and analysed the data to determine the meaning structures that describe the phenomenon of *enabling recovery following peer‐perpetrated sexual violence as it is experienced by young women*. A phenomenological analysis was conducted in accordance with the methodological principles of RLR [15]. The intention of the analysis was to describe the essential meaning of the phenomenon under study and its constituents. An essential meaning is a structure of meanings that describes the phenomenon in a significant way. The analysis process can be understood as a movement between the whole (interviews) and the parts (meaning) as well as a new whole (essential meaning).

Firstly, all the interviews were read without preconceptions to grasp the lived experience of the participants and to open the researchers' minds to the possible meanings in the data. This was done to acquire an overall understanding. The analysis continued by searching for meanings. Meanings that seemed to belong together were grouped into clusters. The analysis was continued by looking for how these clusters were interconnected in a search for patterns of meanings. We moved between the parts and the whole to gain a deeper understanding of the phenomenon and to describe its essential meaning structure. Patterns and shifts in meaning gradually formed into the phenomenon's otherness. This otherness refers to something new, something not already known about the phenomenon [17]. After this description, the analysis process continued, seeking to identify variations and nuances within the phenomenon. Continuously shifting between the whole and the parts, clusters of meaning and the interviews, helped to maintain a bridling stance towards the phenomenon. Bridling one's understanding of the phenomenon enables one to reflect upon and question the findings, thereby allowing its essential meaning to emerge. The essential meaning provides a new description of the phenomenon, and, together with its constituents, which describe variations in the essential meaning, it contributes to a new understanding of the phenomenon. The results section first presents the essential meaning and then describes the meanings that further constitute the phenomenon, with its variations and individual nuances. The phenomenon in question is: *enabling recovery following peer‐perpetrated sexual violence*.

### 2.3 Ethical Considerations

This study was approved by the Swedish Ethical Review Authority 2023‐06‐04 (Dnr 2023‐02464‐01), and we followed the ethical standards of the Helsinki Declaration [18]. Given the sensitivity of the topic, participants' identities were protected and confidentiality was maintained. Verbal and written information about the study and its aim was provided to all participants before their interview. To allow all participants sufficient time to consider their participation in the study, a minimum of 14 days elapsed between initial contact and the interview. Informed consent for participation in this study was obtained verbally. Recorded verbal consent is permitted by the Swedish Ethical Board and was preferred because being subjected to sexual violence is considered sensitive personal data, and verbal consent made it possible to minimise the amount of personal data collected.

## 3 Results

### 3.1 The Essential Meaning

The essential meaning of enabling recovery following peer‐perpetrated sexual violence is described by young women as: by unveiling the hidden, the movement of life reclaims existence.

The essence of enabling recovery following peer‐perpetrated sexual violence can be described as the movement that provides life with meaning and purpose once more. When the hidden is unveiled and existence is reclaimed, a person regains the ability to engage in meaningful and enriching activities that provide life with meaning and purpose and contribute to the rewriting of their story. The unveiling movement begins when an insight strikes the young women and an understanding of the abuse starts to emerge. When the hidden begins to unveil, it is described as moments of clarity and a movement between pain, acceptance and sorrow. This movement contributes to enabling recovery by making the young women's own existence and value visible to them. The process of sharing one's story in a safe place illuminates a way to remake the self and take charge of one's life. Seeing their own value and existence gives them clarity, and they begin to change how they view themselves and their experience of the abuse. The abuse divides life into a before and after, disrupting the movement of life such that the former self no longer exists. The ability to gain trust in the surrounding world is pivotal for enabling recovery following peer‐perpetrated sexual violence.

Stillness and interpersonal connection are required in order to reclaim existence, because they offer opportunities for rest and to gain distance from the intrusive, embodied experience that peer‐perpetrated sexual violence is. For trust and connection to emerge once again after the abuse, restoring existential safeness is significant. Existential safeness can be described as a deep bodily sense of safety that brings one's inner and outer reality into alignment and reconnects one to a sense of inner stillness. The rise of existential safeness illuminates a new beginning in which recovery can be enabled, the ability to engage in meaningful activities is regained and life can begin to move forward again.

The essential meaning is further described by four constituents: *Striking insight*, *A haven for rest*, *Existential safeness* and *Sharing one's story in a safe place*.

### 3.2 Constituents

#### 3.2.1 Striking Insight

Striking insight refers to a moment when a deep understanding of what has occurred becomes visible. This moment of insight can be built up from fractions over time or appear suddenly when sharing thoughts together with someone, but it can also be something that one experiences within oneself.

It just came crashing down on me like…well… a bolt of lightning. That was the first time I thought he had raped me. (2)

A new clarity about oneself emerges, evoking a sense of vulnerability and the insight contributes to the emergence of unveiled emotions and memories. It is important to understand what has happened before recovery can be enabled because embodied memories can be hidden deep within oneself to protect against painful awareness.

…I was seeing a midwife for something else…//…and then she asked if I've ever experienced sexual violence and it hit me—like, yes, I actually have…// I burst into tears, and it was chaos. Up until then, it was as if my mind was a total blank…(10)

This striking insight contributes to the understanding and acceptance of the emotions and bodily reactions that occur in the aftermath of peer‐perpetrated sexual violence. The guilt and shame once directed inward begin to fade when an understanding of the abuse starts to emerge.

…but now I've realised it happened and it really sucked, it was really tough. And I'm not the only one, there are so many, all ages, from different countries and there's no common thread, it just happens to anyone and there's nothing wrong with me. (8)

Yes, tune in to yourself, and actually being in my body has been hard…// It's easier today, I don't think showering is hard anymore like it used to be. (10)

The insight disrupts a person's isolation and detachment from existence, and it is described by the young women as a sense of freedom.

It was such a feeling of freedom, it felt like coming out of a cage… (5)

The striking insight can enable the development of a sense of capacity, and experiencing oneself as capable, as having survived something, creates a sense of inner strength and, eventually, resilience. Resilience strengthens a sense of belonging and connection to oneself, to others and to the surrounding world, and therefore contributes to the movement of life accelerating again.

#### 3.2.2 A Haven for Rest

A haven for rest designates a sanctuary, a place of stillness where one can dwell in tranquillity. This place can be found both inside oneself and together with others. A haven for rest is described as a place that allows one to escape from intrusive thoughts, fear and embodied memories. Stillness can also mean being active and can involve movement, while simultaneously being described as a form of rest in the present moment, where thoughts and emotions coexist in harmony, experienced as a sense of comfort and well‐being. Stillness also provides a break from intrusive memories, fear and loneliness. Creating moments of stillness enables recovery because it provides opportunities for rest and well‐being. The ability to create moments of stillness is crucial, as they are a way to gather strength and provide distance from the ongoing reality.

The hours I was away dancing…the music was so loud that I couldn't hear my thoughts. I was—my mind felt completely at ease…(7)

To dwell in tranquillity can also mean being illuminated by stillness and being able to express one's emotions without words. Creating moments of stillness alone provides opportunities for reflection, allowing one to process thoughts and feelings. Being creative, therefore, becomes a sanctuary where rest and well‐being are possible. Walking by the shore, watching the ocean, taking care of one's pet or being focused on writing in a diary, creating music or crafting are described as self‐created moments of stillness that provide time for self‐reflection.

It felt as though, when I was writing, I was able to let everything out, and in doing so…it was as if I was processing it gradually…(8)

These moments enhance the sense of stillness, tranquillity and well‐being. When engaging in writing, they also provide the necessary language and give words to experiences that are difficult or impossible to talk about. It therefore becomes important for the young women to have access to multiple ways to express themselves before they are ready to share their stories with others.

It became my way of processing and healing because I felt a sense of strength when I was writing. (10)

Trustful relationships, togetherness and an inner sense of existential safeness make it possible to create havens together and within oneself. If the young women had difficulties creating moments of stillness, they might turn to alcohol, drugs or other self‐destructive or self‐harming behaviours to obtain the distance and rest they needed from anxiety, intrusive thoughts and memories.

#### 3.2.3 Existential Safeness

Existential safeness is a fundamental sense that can be understood as a deep, embodied feeling of safety that encompasses emotional, physical and social dimensions. It is significant for enabling recovery.

When relationships are characterised by a supportive and accepting shared existence where no judgement or evaluation is present, a deep embodied feeling of safety is created. This can be described as existential safeness. This means that, when one's existence is acknowledged and protected by others, existential safeness can grow, and meaningful relationships become possible. Existential safeness restores one's dignity, leading to self‐compassion and the ability to feel connected with others. Feeling safe with others requires trust, compassion and acceptance, which lead to a sense of being cared for and being cared for provides a sense of belonging and connection.

…but having a secure network around me made a big difference, too. People I could spend time with, so that I didn't end up isolating myself at home… instead, I went out into the world and dared to do things, even though I was afraid that something might happen or having a hard time feeling safe. (10)

Being subjected to peer‐perpetrated sexual violence takes away one's profound trust in others and isolates individuals in a loneliness where other people are perceived as dangerous. Therefore, trust is significant for enabling recovery because it makes life meaningful and manageable again.

Existential safeness can create insight into one's value, and a sense of significance emerges. Feeling significant implies being alive, being important and recognising oneself as someone who matters, not only to oneself but also to others. Life is made possible when trust and togetherness are present: a profound connection in which sharing a sense of unity and closeness is essential. It also embodies the end of disconnection from others and the surrounding world. Trust can also lead to new intimate relationships, creating opportunities for new embodied memories.

I wanted to keep living and to keep feeling that there really are things worth living for, that I matter here, that I need to be alive. //…and just to experience this… that someone could actually like me. (9)

When existential safeness is present, it means that one's existence has meaning and purpose, revealing an inner sense of value. When one feels valuable to others, a new form of kindness emerges, directed towards oneself. Caring for oneself enables recovery, as a sense of forgiveness begins to emerge. Recovery can also be enabled when one feels responsible for someone else.

During the pregnancy, I knew that I was now responsible for another life. And then I can't hurt myself, because I'd be hurting this little life that I love more than anything I've ever loved. // …I can't miss out on her… // I have to be there with her. (9)

When a sense of obligation emerges, it can provide a reason to strive for survival. In this way, existential safeness contributes to new strategies for life by creating opportunities to reveal strengths and diverse approaches to resilience among young women.

#### 3.2.4 Sharing One's Story in a Safe Place

Sharing one's story in a safe place means sharing experiences of the peer‐perpetrated sexual violence within a place where trust and togetherness are present. When experiencing togetherness with someone, painful memories can be expressed and the suffering caused by the abuse is recognised without judgement.

She said she couldn't offer treatment, but she could listen… //…and it felt like she truly saved my life. (2)

Sharing one's story in a safe place ends the silence surrounding abuse, and this enables recovery. Sharing one's experiences helps to organise thoughts and can sometimes uncover hidden memories. These memories gradually emerge when sharing them offers new perspectives on what happened, but it also provides an opportunity to take control of the narrative.

That I needed to take charge of history, and the more I talked about it, the more it became apparent what had really happened that I'd suppressed for many years. (1)

Sharing one's story requires the ability to shape thoughts and emotions into words. When language is developed around sexual violence, it offers a means to regain control over the situation, both physically and emotionally. This can create opportunities for a deeper understanding of one's circumstances.

…in fact, I had a great need to…er…I felt an urge to tell, and I think it helped me a lot just to be able to express everything in words…(6)

…because when you put these fuzzy words on it made it more manageable, like I understood and had control over the situation. (3)

When the story is acknowledged in a safe place, it reduces feelings of guilt and shame. However, if the story is questioned and the listener lacks the capacity to create a safe space, it evokes a sense of being violated again. Each time the story is shared, it involves re‐experiencing the violence. However, feeling validated when sharing one's story fosters a sense of strength and capacity, thereby breaking the silence. Sharing one's story in a safe place is described as an act of resistance, and when resistance becomes possible, it enables recovery by allowing individuals to regain ownership of their lives. This opens up new opportunities for reinventing oneself after being subjected to peer‐perpetrated sexual violence.

### 3.3 Methodological Reflections

This study has explored the meaning of enabling recovery following peer‐perpetrated sexual violence as it is experienced by young women in Sweden. The study employed a reflective lifeworld research (RLR) approach. Data collection involved 11 online meetings and one telephone interview. Recognising the subject's sensitivity and the geographical diversity of participants, these methods enabled participant engagement within a neutral and safe environment. While lifeworld interviews are typically conducted in person to establish closeness [15, 19], the digital format was deemed suitable given participants' familiarity with online communication. A study by Thunberg and Arnell [20] found that vulnerable groups prefer online meetings when discussing sensitive topics because they perceive them as beneficial, particularly for those facing challenges such as distance or disability. Although online formats can be scrutinised, the interviews resulted in rich descriptions of the phenomenon. RLR requires bridling oneself through openness and flexibility regarding the phenomenon. This was achieved through constant questioning and reflection upon the findings and meanings uncovered during the analysis.

The context of this study should be acknowledged. All the participants were native Swedes and thus constituted a homogeneous group. Including women from diverse ethnic backgrounds, trans women and non‐Swedish speakers in Sweden could have provided deeper insights. Phenomenological findings are presented as essential meanings supported by their constituents, thereby enhancing their generalisability [19]. Nevertheless, phenomenological results should always be understood within their unique contexts [15, 19].

## 4 Discussion

This study has described the meaning of enabling recovery following peer‐perpetrated sexual violence as it is experienced by young women. The essential meaning provides a new description of this phenomenon and, together with its constituents, which describe variations in the essential meaning, it contributes to a new understanding of the phenomenon. Recovery is enabled following peer‐perpetrated sexual violence through the ability to make the disrupted life movement accelerate again. This disrupted life movement is expressed by young women through isolation, silence and a deep sense of emptiness [14]. Therefore, the restoration of life movement is how recovery is enabled following peer‐perpetrated sexual violence.

The movement of life is a dynamic, interconnected movement shaped by multiple elements that simultaneously influence each other. The movement is not linear or isolated but driven by interactions between the four constituents, described here as: striking insight, a haven for rest, existential safeness and sharing one's story. This essential meaning reveals that, for recovery to be enabled following peer‐perpetrated sexual violence, the hidden needs to be uncovered before the movement of life can accelerate again and existence can be reclaimed.

In psychiatric research, recovery can be described as “reconnecting with oneself while struggling between life and death” ([21, p. 202]). After being subjected to peer‐perpetrated sexual violence, young women are fighting to regain their existence. The core of existence is that *existence precedes essence*: humans first exist, then create themselves [22]. When humans are viewed as active beings capable of goal‐oriented action who respond intelligently to their environment, this is understood as an essential feature of our embodied existence [23]. Similarly, reconnection can be understood as a movement that connects oneself to existence once more. For this movement to progress into motion, the young women needed to achieve the insight that someone had done them wrong and that they were not responsible for the sexual violence that occurred. This insight could consist of a moment of clarity or develop slowly as fractions were assembled over time. To understand the movement by which such insight occurs, Cahill's [7] theory on sexual violence can be useful.

Cahill argues that both the social and political environment will affect women's views on the sexual violence to which they have been subjected. This means that the prevailing surroundings can affect young women's views of peer‐perpetrated sexual violence and can contribute to making their individual experiences invisible. The current environment in society will also affect their view of the perpetrator and how public institutions like healthcare will define and react to the sexual violence [7]. Cahill [7] further argues that sexual violence has a cultural meaning which reinforces social structures within society because it is a part of the ongoing history of sexual violence against women by men. This implies that, for a young woman who is subjected to peer‐perpetrated sexual violence by a male acquaintance, this historical consciousness will be present. The experience of being a young woman includes the fear of being raped [7]. This means that young girls and women learn early in life to adapt their movements in the world, with the intention of taking responsibility for their own protection. If a young woman is subjected to sexual violence or sexual harassment, she will feel responsible for the situation and shame and guilt will be a part of the experience [7, 8, 24]. This suggests that young women with a history of peer‐perpetrated sexual violence have to resist societal, historical and individual beliefs before recovery can be enabled. When a person is viewed as an object available for someone else's use, a fragmentation of one's existence appears, in which a sense of alienation and isolation arises [12]. Young women can no longer identify themselves with the person they were before experiencing peer‐perpetrated sexual violence, nor can they envision their future self, leading them into an existential void where their life movement is disrupted [14].

To make life bearable, the young women created havens for rest. This was achieved by engaging in activities that provided stillness for them. Stillness can be understood as a form of rest where a sense of well‐being is present. Stillness can also be active and involve physical movement [12]. The young women turned to creativity, writing, music and group activities to find respite, to give themselves rest from the ongoing violence in their embodied memories. A sense of being caged in the emotional aftermath arose after the peer‐perpetrated sexual violence, but when the young women created havens for rest, a sense of freedom emerged. This can be understood as a capacity to engage in activities that provided stillness in their lives and gave them rest from intrusive memories. When the young women created havens for rest, they also enhanced their sense of well‐being. This capacity to create places for stillness and well‐being is a crucial finding, because it implies that enhancing well‐being can be a valuable approach to enabling recovery when caring for young women with a history of peer‐perpetrated sexual violence. This is significant because, when well‐being influences life, it provides positive opportunities and serves as a resource, independent of health or illness [25]. Galvin and Todres [12] argue that humans recognise well‐being in many different forms and nuances when it is present, and also recognise its absence when suffering. There are several different kinds and levels of well‐being available to human existence, and well‐being and suffering always exist in relation to one another [12].

The results also identified existential safeness as an important element for enabling recovery. Existential safeness can be described as both an embodied feeling of being safe in the world and a sense of alignment that reconnects one's inner and outer reality in stillness. Existential safeness allowed the young women to reconnect with themselves, enabling them to regain control over their lives and making it possible for trustful relationships to develop. Being able to develop trustful relationships was crucial because it restored the young women's trust in others and in themselves. It is important to understand this when caring for young women who have experienced peer‐perpetrated sexual violence, because a lack of existential safeness can hinder the movement of life, and healthcare professionals have a unique opportunity to support the emergence of existential safeness. Galvin and Todres [12] argue that humanly sensitive care requires both a perspective that focuses on well‐being as a direction for care and an understanding of suffering, where suffering announces vulnerability and well‐being announces freedom. This dual understanding is essential because it acknowledges both the individual's vulnerabilities and their possibilities for attaining freedom. When existential safeness is present in young women's lives, their sense of well‐being is strengthened and freedom becomes possible. This opens up opportunities to form trustful relationships and thereby enables recovery following peer‐perpetrated sexual violence.

Sharing one's story was another element that enabled recovery. The ability to develop the necessary language to conceptualise one's experiences creates opportunities for a deeper understanding of oneself. It also provides opportunities to disclose the sexual violence. The disclosure of peer‐perpetrated sexual violence requires a certain sensitivity from healthcare professionals. When the young women felt validated and their story was acknowledged, this made it possible for them to regain ownership over the story and over their lives. Therefore, sharing one's story was particularly important for the young women during the process of reinventing themselves following peer‐perpetrated sexual violence. Previous research supports our findings with similar results. Brison [24] argues that the process of remaking oneself after experiencing sexual violence includes telling one's story and the experience of caring for, and being cared for by, others. Herman [8] describes how traumatic events fundamentally destroy the belief that one can exist in relation to others. These kinds of events, like peer‐perpetrated sexual violence, contribute to the sense that one has been extinguished. And, in some sense, the self only exists in relation to others [8]. Cahill [7] describes sexual violence as an experience of the embodied subject. She argues that all subjects are embodied, but all subjects are embodied differently. Every individual's embodiment is influenced by their gender, race, sex, sexual orientation, historical location and social environment. This means that any attempt to construct a unification of the experience of sexual violence would fall short [7].

When caring for young women with a history of peer‐perpetrated sexual violence, the aim should be to support them in restoring their life movement, because this enables recovery, and strengthens health and well‐being. The young women strive towards well‐being through engaging in movement, stillness and storytelling and by grasping their new reality, and together these interwoven elements of movement create possibilities for enabling recovery.

The results of this study show that healthcare professionals can support young women in enabling recovery by helping them to acknowledge the abuse and by giving them the language to describe their experiences. They should encourage young women with a history of peer‐perpetrated sexual violence to continue engaging in the sports and hobbies that matter to them, and to take part in creative activities that offer moments of stillness and self‐reflection. Healthcare professionals can also create space for disclosure, validate the suffering that emerges after sexual violence and assist the young women in reclaiming their story at a pace that feels safe. Supporting them in sharing their experiences with important people, such as family members or friends, can strengthen their social network and help to rebuild trusting relationships. A healthcare professional may be the first person to initiate a trusting relationship by providing a safe, non‐evaluative space where existential safety can emerge and where their story can unfold without blame or judgement.

Wiklund Gustin [26] emphasises the need to shift care from procedural planning to mutual involvement between healthcare professionals and patients in order to enable recovery. Caring involves supporting and strengthening health and well‐being, even when existence is threatened by illness [12]. Dahlberg, Todres and Galvin [13] further highlight the importance of recognising lived experiences in caring situations, because this promotes well‐being. Such recognition is achieved when healthcare professionals encourage self‐reflection and shift their mindset from merely doing care to truly being caring. It also involves engaging with the patient's lifeworld and acknowledging the patient as the expert on their own situation.

## 5 Conclusion

The contribution of this study is to provide a complex and nuanced understanding of how recovery is enabled following peer‐perpetrated sexual violence. Young women's health and well‐being are impacted over time, highlighting the importance of prioritising a lifeworld‐led, recovery‐oriented caring approach rooted in caring science, in which both physical and existential dimensions are acknowledged. The findings emphasise the importance of uncovering the experience of peer‐perpetrated sexual violence to enable the ability to reclaim one's existence, where existential safeness and stillness are fundamental. Every element of enabling recovery is intertwined, with no part functioning in isolation. It is therefore essential to understand recovery as a dynamic movement of meaning‐making that contributes to health and well‐being.

## Author Contributions

J. Isaksson contributed to study planning, data collection and analysis, and led the manuscript writing. L. Palmér and M. Lundvall have contributed to data analysis, manuscript writing, revision and supervision. All authors have reviewed and approved the final manuscript.

## Funding

This paper was funded by the University of Borås.

## Ethics Statement

This study received ethical approval from the Swedish Ethical Review Authority 2023.06.04.

## Conflicts of Interest

The authors declare no conflicts of interest.

## Data Availability

Data material is stored at the University of Borås and may be made available upon reasonable request, subject to ethical considerations.
